# Sexual dysfunction and satisfaction in women in the late postpartum period: a correlational study

**DOI:** 10.1590/0034-7167-2023-0144

**Published:** 2025-03-14

**Authors:** Tayenne Maranhão de Oliveira, Josefa Nayara de Lima, Rachel de Sá Barreto Luna Callou Cruz, Antonio Germane Alves Pinto, Luis Rafael Leite Sampaio, Glauberto da Silva Quirino

**Affiliations:** I Centro Universitário Paraíso. Juazeiro do Norte, Ceará, Brazil; II Universidade Regional do Cariri. Crato, Ceará, Brazil

**Keywords:** Postpartum Period, Sexual Behavior, Sexuality, Sexual Dysfunction, Physiological, Primary Health Care, Periodo Posparto, Conducta Sexual, Sexualidad, Disfunciones Sexuales Fisiológicas, Atención Primaria de Salud

## Abstract

**Objectives:**

to analyze sexual dysfunction in women in the late postpartum period and correlate it with sexual satisfaction.

**Methods:**

this is a cross-sectional study conducted with 351 women in the late postpartum period, linked to the Family Health Strategy. Three questionnaires were used for data collection. Inferential analysis was performed using Pearson’s correlation and Student’s t-tests.

**Results:**

the prevalence of dysfunction was 96.2%, and no statistically significant difference was found when considering the mode of delivery (p>0.05). Sexual dysfunction was present in all domains of sexual function (p<0.05), and a significant difference was observed between sexual satisfaction scores and the presence of dysfunction (p<0.05).

**Conclusions:**

there was a high prevalence of sexual dysfunction in the late postpartum period, with no correlation to the mode of delivery. The dysfunction affected all domains of sexual function and negatively influenced women’s sexual satisfaction.

## RESUMEN

**Objetivos:**

analizar la disfunción sexual en mujeres en el puerperio remoto y correlacionarla con la satisfacción sexual.

**Métodos:**

estudio transversal realizado con 351 mujeres en el puerperio remoto, vinculadas a la Estrategia Salud de la Familia. Se utilizaron tres formularios para la recolección de datos. El análisis inferencial se realizó mediante las pruebas de correlación de Pearson y t de *Student*.

**Resultados:**

la prevalencia de disfunción fue del 96,2%, y no hubo diferencias estadísticamente significativas al considerar la vía de parto (p>0,05). Se constató la presencia de disfunción sexual en todos los dominios de la función sexual (p<0,05) y una diferencia significativa entre los puntajes de satisfacción sexual y la presencia de disfunción (p<0,05).

**Conclusiones:**

hubo una alta prevalencia de disfunción sexual en el puerperio remoto, sin correlación con la vía de parto. La disfunción afectó todos los dominios de la función sexual e influyó negativamente en la satisfacción sexual de las mujeres.

## INTRODUCTION

Historically, female sexual function has been conceptualized in terms of a woman’s reproductive capacity and her social role as a mother, a conception that has increasingly been questioned, as there is a need to focus on women’s sexual and emotional needs^(1)^.

From this perspective, sexual function represents an important element in women’s health and quality of life, and it can be influenced by the interaction of physical, social, behavioral, and cultural factors. Taking these factors into account, the model of female sexual function proposes a cycle of overlapping domains based on subjective experience and physiological changes: desire, pain, arousal, lubrication, and orgasm^(2)^.

In the context of sexual function, a central issue is sexual satisfaction, as there is a subtle connection between the two, supported by data that show significant positive correlations between sexual satisfaction and female sexual function^(3)^. Therefore, addressing satisfaction in this context becomes even more significant when considering that a satisfying sex life positively impacts self-esteem, well-being, and overall quality of life^(4)^.

In this sense, any alteration, whether psychogenic and/or organic in origin, in one or more domains of sexual function is classified as sexual dysfunction, encompassing issues with desire, arousal, lubrication, orgasm, and/or sexual pain^(5)^. Although the causes are multifactorial, mental health impairment emerges as the most important risk factor for female sexual dysfunction^(2)^. As such, these dysfunctions depend on a range of personal and emotional stimuli, and women in the pregnancy-postpartum cycle may experience adverse outcomes in their sexual function^(6)^.

The pregnancy-postpartum cycle affects various aspects of a woman’s life due to anatomical, physiological, psychological, and social changes, which, combined with other specific demands of this period, such as caring for a newborn and sleep deprivation, negatively impact sexual function and increase sexual dysfunction in women, especially during the postpartum period^(7,8)^.

The literature points to a high prevalence of sexual dysfunction in the postpartum period, as evidenced by two studies conducted in Spain and China, where sexual dysfunctions were observed in 89.7% and 97% of the women evaluated, respectively^(9,10)^. In Brazil, there is a scarcity of data on the prevalence of sexual dysfunction in the postpartum period. However, a study published in 2018 found that 78.2% of participants experienced sexual dysfunction^(11)^.

Sexual dysfunction predominantly begins in the late postpartum period^(9)^, which starts on the 43rd day after childbirth, and is associated with variables such as breastfeeding, depressive symptoms, partner quality, thirdand fourth-degree perineal trauma, postpartum weight gain, and the use of hormonal contraception^(12-14)^.

However, although the high prevalence is recognized, the problem still requires greater visibility, as aspects related to sexual function continue to be neglected during the pregnancy-postpartum cycle. For this reason, it is necessary to develop actions to encourage and provide counseling on sexual activity, particularly in the late postpartum period, when sexual practices typically resume^(12,13)^.

This issue should be a target of intervention, as sexual dysfunctions can interfere with female sexual satisfaction, which is an essential component of sexual health and an indicator of quality of life and well-being, being strongly associated with positive sexual experiences^(15)^.

In this regard, the production of knowledge related to female sexual satisfaction and dysfunction is indispensable for the development of practices in the context of nursing care, as the goal is to develop actions directed at women’s health with a focus on integrality. Moreover, addressing issues that permeate sexual health in its various aspects is a fundamental competency for nurses^(16)^.

Given the scarcity of national data on sexual dysfunction in the late postpartum period and the growing interest in sexual satisfaction as an important component of quality of life, it is necessary to identify and highlight the interaction between sexual dysfunction and female sexual satisfaction to develop appropriate nursing care.

## OBJECTIVES

To analyze sexual dysfunction in women in the late postpartum period and correlate it with sexual satisfaction.

## METHODS

### Ethical aspects

The research was conducted in accordance with national and international ethical guidelines and was approved by the Research Ethics Committee of the Regional University of Cariri. In addition, written informed consent was obtained from all participants involved in the study.

### Study design, period, and location

This is a cross-sectional, correlational study that followed the Strengthening the Reporting of Observational Studies in Epidemiology (STROBE) guidelines. It was conducted within the area covered by 67 Family Health Strategy Teams in the city of Juazeiro do Norte, Ceará, Brazil. Data collection took place between April and September 2018.

### Population or sample; inclusion and exclusion criteria

The prevalence of sexual dysfunction during the puerperium was estimated based on a median prevalence of 52%, as indicated by a previous study for this period, which ranged from 18% to 86%^(17)^. The sample size was calculated using the formula for finite populations. The parameters considered were a confidence level of 95%, an expected accuracy of 52%, an expected error of 48%, a total population of 4,069 (corresponding to the number of deliveries performed annually in the municipality where the research was conducted), and a precision level of 5%, resulting in a sample of 351 women.

Women were included if they were in the late postpartum period (at least 70 days after delivery) and were residents of Juazeiro do Norte. The cutoff point of 70 days was chosen based on the assumption that sexual activities generally resume after 42 days postpartum^(18,19)^.

Women were excluded if they had clinical and/or obstetric complications that contraindicated sexual activity, such as placenta previa, premature rupture of membranes, preterm labor, pregnancy resulting from rape, fetal death, hypertensive disorders of pregnancy, and postpartum hemorrhage, as these complications may subjectively interfere with a woman’s decision to resume her sexual life after childbirth^(10)^.

The women were selected through non-probabilistic convenience sampling, and all those who met the pre-established inclusion criteria were consecutively invited to participate in the study as they were contacted by the nurse from the Family Health Strategy Team to which they were linked. This contact occurred during pediatric follow-up appointments and while administering their children’s immunizations. In addition, home visits were conducted through prior scheduling. Data collection took place at the Basic Health Units in rooms appropriate for this activity and, in some cases, at the participants’ homes.

To minimize voluntarism and other types of bias in sample selection, a sequential and/or consecutive sampling method was used, consisting of the continuous recruitment of all individuals accessible during the study period. Addressing bias is fundamental to ensuring the internal validity of the research^(20)^.

### Study Protocol

Three questionnaires were used for data collection. The first, developed by the researchers, contained sociodemographic variables: age, marital status, education, race/ethnicity, civil status, occupation, type of prenatal care, household income, living conditions, and religion; and obstetric variables: start of prenatal care, number of pregnancies, parity, number of abortions, type of delivery, and gestational age at delivery.

The second instrument was the Female Sexual Function Index (FSFI)^(21)^, which assessed the sexual function of the women. The FSFI was the first instrument for evaluating female sexual function validated in Portuguese^(22)^. It consists of 19 questions, divided into six domains: desire, arousal, lubrication, orgasm, satisfaction, and pain. Individual scores were obtained by summing the items that comprised each domain, called a simple score. The simple score was multiplied by the factor corresponding to that domain, resulting in the weighted score. The final score was obtained by summing the weighted scores for each domain, ranging from 2 to 36. A cutoff score of 26.5 was used to characterize good sexual function; thus, a higher score was associated with lower severity of sexual dysfunction^(21)^.

The third instrument used was the New Sexual Satisfaction Scale (NSSS), which assessed sexual satisfaction. The NSSS version was validated in Portuguese and has strong psychometric properties, consisting of 20 items with a bidimensional factor structure composed of two subscales that evaluate aspects related to the self (self-centered) and the partner and sexual activity^(23)^. Additionally, there were five response options (1 = not at all satisfied, 2 = somewhat satisfied, 3 = moderately satisfied, 4 = very satisfied, 5 = extremely satisfied). The scores for each dimension were obtained by summing the individual item scores within that dimension, and the total NSSS score was obtained by summing the scores of all the items. The higher the score on the scale, the greater the level of sexual satisfaction.

### Analysis of results and statistics

The data were entered, processed, and stored in a database using Microsoft Excel version 7.0 and Microsoft Office Word version 2010. Data organization was conducted through double entry and subsequent validation to control for potential errors during data transcription. The database was then transferred to RStudio software (version 386 3.2.4) for data grouping, organization, and analysis, which included descriptive statistics for frequencies, measures of central tendency, and dispersion, as well as inferential statistics for correlation between variables and the creation of tables with the obtained results.

The prevalence rate of sexual dysfunction among participants was calculated by dividing the number of existing cases by the total study population, then multiplying by 100 to obtain a percentage.

For the statistical analysis, absolute (n) and relative (%) frequencies were used for qualitative variables. The Shapiro-Wilk test was employed to identify the distribution of variables, confirming the normality of the data to be analyzed.

Pearson’s correlation test was used to determine the correlation between sexual function domains and the presence of dysfunction. Student’s t-test for independent samples was applied to compare sexual satisfaction according to the presence of sexual dysfunction. To adjust for possible confounding factors and effect modifiers, the chi-square test was used to compare sexual dysfunction according to the mode of delivery (vaginal or cesarean). A p-value of <0.05 was considered statistically significant.

## RESULTS

The mean age of the participants was 27.3 years, with a minimum age of 14 and a maximum of 43 years. Of the total, 83.8% of the women had basic education; 58.4% self-identified as Black; 63.3% were in some form of union; 41.3% had an income of up to one minimum wage. However, 60.1% reported not working outside the home, and 94.0% followed religions based on Christian doctrines. The sociodemographic data of the participants are presented in Table 1.

**Table 1:** Sociodemographic characteristics of women in the late postpartum period, Juazeiro do Norte, Ceará, Brazil, 2018

Characteristics	n	%
Education Incomplete elementary education Complete elementary education Incomplete high school Complete high school Incomplete higher education Complete higher education Race White Black Brown Asian Works outside the home Yes No Living situation With family members With partner Alone Others Religion Atheist Spiritist Afro-Brazilian Religions Evangelical Catholic None Income Less than one minimum wage One minimum wage More than one minimum wage	27 52 12 203 6 51 103 33 172 43 140 211 145 189 12 5 2 5 4 51 279 10 42 1.2 207	7.7 14.9 3.4 57.8 1.7 14.5 29.3 9.4 49.0 12.3 39.9 60.1 41.3 53.9 3.4 1.4 0.6 1.4 1.1 14.5 79.5 2.9 12.2 29.1 58.7

Regarding obstetric characteristics, 88.6% of the women received prenatal care through the Unified Health System (SUS in Portuguese), and 11.4% used private healthcare. Of the total, 50.4% were primiparous, and the number of pregnancies ranged from one to nine, with an average of 1.7 pregnancies per participant.

In terms of the prevalence of sexual dysfunction, the rate found in this study was 96.2%, with no statistically significant difference in relation to the mode of delivery (p>0.05), indicating that the presence of dysfunction was independent of the type of delivery (Table 2).

**Table 2:** Prevalence and comparison of sexual dysfunction in women in the late postpartum period by mode of delivery, Juazeiro do Norte, Ceará, Brazil, 2018

Type of delivery^*^	With dysfunction	Without dysfunction	*p* value^^**^^
Cesarean Vaginal Total	230 (96.2%) 108 (96.4%) 338 (96.2%)	9 (3.8%) 4 (3.6%) 13 (3.8 %)	1

* No missing data were found;

** Chi-square test.

Upon examining the sexual function domains affected by sexual dysfunction, moderate to strong, positive, and significant correlations (p<0.01) were observed, indicating the presence of sexual dysfunction across all domains, with the lowest scores in the domains of pain and lubrication (Table 3).

**Table 3:** Types of sexual dysfunction identified in the late postpartum period, Juazeiro do Norte, Ceará, Brazil, 2018

Domains of female sexual function^*^	Median FSFI score		*p* value^**^
	Without dysfunction	With dysfunction	
Desejo Excitação Lubrificação Orgasmo Dor	3.6 3.0 2.0 2.5 1.5	1.2 1.2 1.2 1.2 1.2	<0.01 <0.01 <0.01 <0.01 <0.01

* Não houve dados faltantes;

** Teste de correlação de Pearson; FSFI - *Female Sexual Function Index.*

Another result observed was the comparison between sexual satisfaction and sexual dysfunction in postpartum women using Student’s t-test. The mean sexual satisfaction score of women with dysfunction was statistically lower than the mean score of women without sexual dysfunction (Table 4). Thus, it was observed that sexual dysfunction reduces the sexual satisfaction of these women.

**Table 4:** Correlation between sexual satisfaction and dysfunction in the late postpartum period, Juazeiro do Norte, Ceará, Brazil, 2018

	Mean Sexual Satisfaction	*p* value^^*^^
With dysfunction Without dysfunction	45.6 62.7	<0.01

* *Student's t-test.*

## DISCUSSION

Regarding the sociodemographic and obstetric characteristics of the studied population, most corresponded to findings reported in other studies on the subject^(6,24,25)^, such as age, education, and parity. However, given the complexity and significance of this issue, it is prudent to explore other properties and variables included in this context, such as the subjective aspects of sexual function.

In this study, a high prevalence of sexual dysfunctions in the late postpartum period was found, corroborating the results of a study that used the FSFI, conducted through an online questionnaire with 78 women between 45 and 180 days postpartum, where 78.2% presented sexual dysfunction^(11)^.

Thus, it is emphasized that female sexual function is influenced by a complex interaction of factors, with the birthing process being one of the most important, due to the various changes that occur during the gestational period, which supports the high prevalence of postpartum sexual dysfunction^(26)^.

The type of delivery was not a determining factor for sexual dysfunction, as it was present regardless of this factor. These findings are consistent with data from a systematic review, which supported the irrelevance of delivery type on female sexual function in the postpartum period^(27)^. However, although no significant relationship was found between the variables, there are associations between instrumental vaginal delivery and anal sphincter injury with increased chances of sexual dysfunction in the first year postpartum^(14)^.

The influence of delivery type on changes in sexual function was also evaluated in another study with women in the late postpartum period, in which no significant differences were found between the groups of women who underwent vaginal delivery compared to those who had cesarean sections^(28)^.

The data also indicated which domains of female sexual function were affected by dysfunction, with all domains being impacted. Similar findings were observed in a study with Japanese couples, in which the scores for arousal, lubrication, orgasm, and pain decreased from one to three months postpartum^(29)^.

The domains of lubrication and pain in women without dysfunction had the lowest median FSFI scores, and no variation was observed in the median scores of women with dysfunction, unlike the findings of a systematic review with meta-analysis, in which dysfunction in the arousal phase was most prevalent, followed by the desire phase^(30)^. Conversely, another study pointed out that the highest prevalence of dysfunction was associated with the presence of dyspareunia^(31)^.

The sexual satisfaction levels of the women in this study were significantly influenced by sexual dysfunction, negatively impacting their lives in terms of sexual function, which deserves attention in the context of comprehensive health care.

The domains of female sexual function are developed in an integrated manner: arousal is linked to desire, orgasm, and lubrication, and this affects pain, while orgasm plays a vital role in satisfaction^(5)^. Thus, although engaging in sexual activity is not a guarantee of satisfaction, two domains of sexual functioning, desire and orgasm, serve as significant predictors of female sexual satisfaction and that of their partners^(32)^.

The maintenance of sexual function and subsequent satisfaction are among the most important aspects of women’s lives, as they are closely related to quality of life^(33)^. However, sexual function in postpartum women’s health care is often neglected, leading to a deficit in care. This may be attributed to the timid and underdeveloped approach to the subject during prenatal and postnatal consultations, whether due to cultural, religious, or even knowledge gaps^(34,35)^.

The approach to this aspect of sexuality should particularly take place within Primary Health Care, as it is considered the gateway to the healthcare system, whose premise is to promote health and prevent complications. Thus, orientation, counseling, and addressing concerns related to sexual dysfunctions should be part of the nursing care plan from prenatal care, even if the woman does not directly express concerns about the issue^(6)^.

Finally, it is emphasized that discussions about sexuality, function, dysfunction, and sexual satisfaction remain a taboo, even among healthcare professionals, perpetuating cultural stereotypes about the roles of mother and woman in a society structured under a patriarchal framework. Therefore, it is essential to expand understanding and discussion of the multiple factors involved in order to improve sexual health care for women in the postpartum period.

### Study limitations

The limitations of this study are related to the non-probabilistic sampling, the absence of multivariate statistical analysis, and the fact that the perspectives of healthcare professionals from the Family Health Strategy teams, as well as the participants’ partners, were not considered.

### Contributions to the field

Given the high prevalence of sexual dysfunction in the late postpartum period found in this study, the role of the healthcare team, especially nurses, in providing care during the pregnancy-postpartum cycle becomes essential. The topic discussed may have implications for nursing care practice, as it provides elements to support the approach to sexual function during nursing consultations.

In this context, nurses can structure their care to meet the identified needs, with a focus on individual consultations, during which sexual function assessments are conducted, factors influencing sexual function are investigated, advice on sexual practices is provided, discussions about female anatomy and physiology take place, and self-awareness is encouraged.

## CONCLUSIONS

The data obtained indicated a high prevalence of sexual dysfunction in the late postpartum period. However, sexual dysfunction was not correlated with the mode of delivery. Sexual dysfunction was present in all domains of sexual function and negatively influenced the sexual satisfaction of women in the late postpartum period.

It is hoped that the data, as well as the discussions and reflections raised in this study, will support nursing care actions aimed at women’s sexual health, considering the aspects of sexual function in the postpartum period as important for quality of life. Furthermore, it is suggested that other studies in this context be developed to enable a better understanding of the problem from the perspective of sexual partners and healthcare professionals.
