# Supplementary material for: Sexual dysfunction and satisfaction in women in the late postpartum period: a correlational study
Source: Rev Bras Enferm. 2025 Mar 14;78(1):e20230144. doi: 10.1590/0034-7167-2023-0144 (PMC11913044; doi:10.1590/0034-7167-2023-0144)
Supplement: Translation

# Translation: Disfunção e satisfação sexual em mulheres no puerpério remoto: estudo correlacional

**DOI:** 10.1590/0034-7167-2023-0144pt

**Authors:** Tayenne Maranhão de Oliveira, Josefa Nayara de Lima, Rachel de Sá Barreto Luna Callou Cruz, Antonio Germane Alves Pinto, Luis Rafael Leite Sampaio, Glauberto da Silva Quirino

**Affiliations:** I Centro Universitário Paraíso. Juazeiro do Norte, Ceará, Brasil; II Universidade Regional do Cariri. Crato, Ceará, Brasil

**Keywords:** Período Pós-Parto, Comportamento Sexual, Sexualidade, Disfunções Sexuais Fisiológicas, Atenção Primária à, Saúde.

## Abstract

**Objetivos:**

analisar a disfunção sexual em mulheres no puerpério remoto e correlacioná-la com a satisfação sexual.

**Métodos:**

estudo transversal realizado com 351 mulheres no puerpério remoto, vinculadas à Estratégia Saúde da Família. Foram utilizados três formulários para a coleta de dados. A análise inferencial foi realizada por meio dos testes de correlação de Pearson e t de *Student*.

**Resultados:**

a prevalência de disfunção foi de 96,2%, e não houve diferença estatisticamente significativa quando se considerou a via de parto (p>0,05). Constatou-se a presença de disfunção sexual em todos os domínios da função sexual (p<0,05) e uma diferença significativa entre os escores de satisfação sexual e a presença de disfunção (p<0,05).

**Conclusões:**

houve alta prevalência de disfunção sexual no puerpério remoto, sem correlação com a via de parto. A disfunção afetou todos os domínios da função sexual e influenciou negativamente a satisfação sexual das mulheres.

## INTRODUÇÃO

Historicamente, a função sexual feminina foi concebida considerando a capacidade reprodutiva da mulher e com base em seu papel social de mãe, uma concepção que vem sendo questionada, uma vez que se deve considerar os aspectos centrados nas necessidades sexuais e emocionais das mulheres^(1)^.

Nessa perspectiva, a função sexual representa um importante elemento na saúde e na qualidade de vida das mulheres, podendo ser modificada pela interação de fatores físicos, sociais, comportamentais e culturais. Considerando esses fatores, o modelo de função sexual feminina propõe um ciclo de domínios sobrepostos baseado na experiência subjetiva e nas mudanças fisiológicas: desejo, dor, excitação, lubrificação e orgasmo^(2)^.

No escopo da função sexual, uma questão central é a satisfação sexual, uma vez que existe uma conexão tênue entre as duas, fundamentada em dados que apontam correlações positivas significativas entre satisfação sexual e função sexual feminina^(3)^. Portanto, a abordagem da satisfação nesse contexto torna-se ainda mais significativa quando se considera que uma vida sexual satisfatória afeta de forma positiva a autoestima, o bem-estar e a qualidade de vida geral^(4)^.

Nesse sentido, qualquer alteração, de origem psicogênica e/ou orgânica, em um ou mais domínios da função sexual configura-se como disfunção sexual, englobando, assim, problemas no desejo, excitação, lubrificação, orgasmo e/ou dor sexual^(5)^. Embora as causas sejam multifatoriais, o comprometimento da saúde mental comporta-se como o fator de risco mais importante para a disfunção sexual feminina^(2)^. Portanto, essas disfunções dependem de uma série de estímulos pessoais e emocionais, e as mulheres no ciclo gravídico-puerperal podem experimentar desfechos desfavoráveis em sua função sexual^(6)^.

O ciclo gravídico-puerperal afeta diversos aspectos da vida da mulher, em decorrência de alterações anatômicas, fisiológicas, psicológicas e sociais, que, somadas a outras demandas específicas desse período, como os cuidados com o neonato e a privação de sono, impactam negativamente a função sexual e aumentam a disfunção sexual nas mulheres, sobretudo no período puerperal^(7,8)^.

A literatura aponta alta prevalência de disfunção sexual no período pós-parto, como evidenciado por dois estudos realizados na Espanha e na China, nos quais verificou-se a presença de disfunções sexuais em 89,7% e 97% das mulheres avaliadas, respectivamente^(9,10)^. No Brasil, há escassez de dados sobre a prevalência de disfunção sexual no pós-parto. Contudo, em estudo publicado em 2018, foi apontada a presença de disfunção sexual em 78,2% das participantes^(11)^.

A disfunção é iniciada predominantemente no puerpério remoto^(9)^, período a partir do 43º dia após o parto, estando associada a variáveis como amamentação, sintomas depressivos, qualidade da parceria, trauma perineal de terceiro e quarto graus, ganho de peso no pós-parto e uso de contracepção hormonal^(12-14)^.

No entanto, embora se identifique a prevalência acentuada, o problema ainda necessita de maior visibilidade, visto que os aspectos relacionados à função sexual ainda são negligenciados durante o ciclo gravídico-puerperal. Em razão disso, é necessário desenvolver ações para encorajar e aconselhar sobre a atividade sexual, sobretudo no puerpério remoto, quando, habitualmente, há o retorno das práticas sexuais^(12,13)^.

Esse fato deve ser objeto de intervenção, pois as disfunções sexuais podem interferir na satisfação sexual feminina, que é um componente essencial da saúde sexual e um indicador da qualidade de vida e bem-estar das pessoas, estando fortemente associada a experiências positivas da sexualidade^(15)^.

Nesse sentido, a produção de conhecimento relacionada à satisfação e disfunção sexual feminina é indispensável para a construção de práticas no contexto do cuidado de enfermagem, uma vez que se busca desenvolver ações direcionadas à atenção à saúde das mulheres pautadas na integralidade. Ademais, a abordagem de questões que permeiam a saúde sexual, em seus mais diversos sentidos, é uma competência fundamental do enfermeiro^(16)^.

Considerando que os dados nacionais sobre a disfunção sexual no puerpério remoto são escassos e que há um crescente interesse pela satisfação sexual como componente importante da qualidade de vida, é preciso identificar e evidenciar a interação entre disfunção e satisfação sexual feminina, para que se possam desenvolver cuidados de enfermagem oportunos.

## OBJETIVOS

Analisar a disfunção sexual em mulheres no puerpério remoto e correlacioná-la com a satisfação sexual.

## MÉTODOS

### Aspectos éticos

A pesquisa foi desenvolvida em conformidade com as diretrizes éticas nacionais e internacionais, sendo aprovada pelo Comitê de Ética em Pesquisa da Universidade Regional do Cariri. Além disso, o Consentimento Livre e Esclarecido foi obtido de todas as participantes envolvidas no estudo, por meio de documento escrito.

### Desenho, período e local do estudo

Estudo transversal, de caráter correlacional, que seguiu os itens do *Strengthening the Reporting of Observational Studies in Epidemiology* (STROBE). Foi realizado na área de abrangência das 67 Equipes da Estratégia Saúde da Família, na cidade de Juazeiro do Norte, Ceará, Brasil. A coleta de dados ocorreu entre os meses de abril e setembro de 2018.

### População ou amostra; critérios de inclusão e exclusão

Foi utilizada como prevalência de disfunção sexual no período do puerpério a mediana das prevalências, que foi de 52%, conforme estudo anterior para este período, o qual variava entre 18% e 86%^(17)^. O tamanho da amostra foi estimado por meio da fórmula para populações finitas. Dessa forma, consideraram-se como parâmetros o nível de confiança (95%), a quantidade de acerto esperado (52%), a quantidade de erro esperado (48%), a população total (4.069) - que corresponde ao número de partos realizados anualmente no município onde a pesquisa foi conduzida -, e o nível de precisão (5%), resultando em uma amostra de 351 mulheres.

Foram incluídas mulheres no período puerperal remoto, com no mínimo 70 dias de pós-parto, residentes na cidade de Juazeiro do Norte. Para a determinação do ponto de corte de 70 dias, considerou-se que o retorno das atividades sexuais geralmente ocorre após 42 dias do parto^(18,19)^.

Foram excluídas puérperas que tiveram intercorrências clínicas e/ou obstétricas que contraindicassem a atividade sexual, como placenta prévia, ruptura prematura de membranas, trabalho de parto prematuro, gravidez resultante de estupro, morte fetal, síndromes hipertensivas da gestação e hemorragia puerperal, visto que essas intercorrências gestacionais têm potencial para interferir subjetivamente na decisão da mulher de retomar sua vida sexual após o parto^(10)^.

As mulheres foram selecionadas por amostragem não probabilística por conveniência, e todas as que atendiam aos critérios de inclusão pré-estabelecidos foram convidadas a participar da pesquisa de forma consecutiva, à medida que eram contatadas pelo enfermeiro da Equipe da Estratégia Saúde da Família, à qual estavam vinculadas. Esse contato ocorria durante as consultas de puericultura e a realização da imunização de seus filhos. Além disso, realizaram-se visitas domiciliares, mediante agendamento prévio. A coleta de dados ocorreu nas Unidades Básicas de Saúde, em sala adequada para essa atividade, e, em alguns casos, no domicílio das participantes.

Para minimizar o voluntarismo e outros tipos de vieses na seleção da amostra, a amostragem foi sequencial e/ou consecutiva, o que consistiu no recrutamento contínuo de todas as pessoas acessíveis durante o período determinado pelo estudo. A abordagem dos vieses é fundamental para garantir a validade interna da pesquisa^(20)^.

### Protocolo do estudo

Três formulários foram utilizados para a coleta de dados. O primeiro, construído pelos pesquisadores, continha variáveis sociodemográficas: idade, estado civil, escolaridade, cor/raça, estado marital, ocupação, tipo de assistência pré-natal, renda familiar, condições de moradia e religião; e variáveis obstétricas: início do acompanhamento pré-natal, número de gestações, paridade, número de abortos, tipo de parto e idade gestacional no momento do parto.

O segundo instrumento foi o *Female Sexual Function Index* (FSFI)^(21)^, que avaliou a função sexual das mulheres. O FSFI foi o primeiro instrumento para avaliação da função sexual feminina validado para o português^(22)^. Ele é constituído por 19 questões, divididas em seis domínios: desejo, excitação, lubrificação, orgasmo, satisfação e dor. As pontuações individuais foram obtidas pela soma dos itens que compunham cada domínio, chamado de escore simples. O escore simples foi multiplicado pelo fator correspondente a esse domínio, resultando no escore ponderado. A pontuação final foi obtida pela soma dos escores ponderados de cada domínio, podendo variar de 2 a 36. O ponto de corte para caracterizar uma boa função sexual era 26,5; assim, uma pontuação mais alta estava associada a menor gravidade da disfunção sexual^(21)^.

O terceiro instrumento utilizado foi a *New Sexual Satisfaction Scale* (NSSS), que avaliou a satisfação sexual. A versão do NSSS foi validada para o português e apresenta sólidas propriedades psicométricas, sendo constituída por 20 itens, com uma estrutura fatorial bidimensional composta por duas subescalas que avaliaram aspectos relacionados ao eu (egocentrado) e ao parceiro e à atividade sexual^(23)^. Além disso, havia cinco opções de resposta (1 = nada satisfeito, 2 = um pouco satisfeito, 3 = moderadamente satisfeito, 4 = muito satisfeito, 5 = extremamente satisfeito). As pontuações de cada dimensão foram obtidas pela soma das pontuações dos itens individuais dessa dimensão, e a pontuação total da NSSS foi obtida pela soma das pontuações de todos os itens. Quanto maior fosse a pontuação na escala, maior seria o nível de satisfação sexual.

### Análise dos resultados e estatística

Os dados foram digitados, processados e armazenados em banco de dados utilizando-se o *Microsoft Excel* versão 7.0 e o *Microsoft Office Word* versão 2010. A organização dos dados foi feita por meio de dupla digitação e posterior validação, a fim de controlar possíveis erros na transposição das informações. O banco de dados foi transferido para o *software RStudio* (Versão 386 3.2.4) para o agrupamento, organização e análise dos dados, com a realização de estatísticas descritivas de frequências, medidas de tendência central e dispersão, além da correlação das variáveis por meio de estatística inferencial e elaboração de tabelas com os resultados obtidos.

A taxa de prevalência de disfunção sexual nas participantes foi calculada de acordo com o número de casos existentes, dividido pelo número total da população estudada, multiplicado por 100, resultando em um percentual.

Quanto à análise estatística, para as variáveis qualitativas, foram utilizadas as frequências absolutas (n) e relativas (%). O teste de Shapiro-Wilk foi utilizado para identificar a distribuição das variáveis, sendo constatada a normalidade dos dados a serem analisados.

O teste de correlação de Pearson foi empregado para determinar a correlação entre os domínios da função sexual e a presença de disfunção. O teste t de Student para amostras independentes foi utilizado para comparar a satisfação sexual de acordo com a presença de disfunção sexual. Com o objetivo de ajustar possíveis fatores de confusão e modificadores do efeito, foi utilizado o teste do qui-quadrado para a comparação da disfunção sexual segundo a via de parto (vaginal e cesárea). Valores de p<0,05 foram considerados significativos.

## RESULTADOS

A média de idade das participantes foi de 27,3 anos, com idade mínima de 14 e máxima de 43 anos. Do total, 83,8% das mulheres tinham educação básica; 58,4% se autodeclararam negras; 63,3% estavam em algum tipo de união; 41,3% possuíam renda de até um salário-mínimo. No entanto, 60,1% afirmaram não trabalhar fora de casa, e 94,0% seguiam religiões baseadas em doutrinas cristãs. Os dados referentes à caracterização sociodemográfica das participantes podem ser verificados na Tabela 1.

**Tabela 1:** Caracterização sociodemográfica das mulheres no puerpério remoto, Juazeiro do Norte, Ceará, Brasil, 2018

Características	n	%
Escolaridade Ensino Fundamental incompleto Ensino fundamental completo Ensino médio incompleto Ensino médio completo Ensino Superior incompleto Ensino superior completo Cor Branca Preta Parda Amarela Trabalha fora Sim Não Com quem mora Familiares Companheiro Sozinha Outros Religião Ateu Espírita Cultos afro-brasileiros Evangélica Católica Nenhuma Renda Menos de um salário-mínimo Um salário-mínimo Mais de um salário-mínimo	27 52 12 203 6 51 103 33 172 43 140 211 145 189 12 5 2 5 4 51 279 10 42 1,2 207	7,7 14,9 3,4 57,8 1,7 14,5 29,3 9,4 49,0 12,3 39,9 60,1 41,3 53,9 3,4 1,4 0,6 1,4 1,1 14,5 79,5 2,9 12,2 29,1 58,7

Em relação ao perfil obstétrico, 88,6% das mulheres realizaram o pré-natal no Sistema Único de Saúde (SUS) e 11,4% na rede particular. Do total, 50,4% eram primíparas, e o número de gestações variou de uma a nove, com uma média de 1,7 gestações por participante.

No que se refere à prevalência de disfunção sexual, a taxa encontrada neste estudo foi de 96,2%, sem diferença estatisticamente significativa em relação à via de parto (p>0,05), indicando que a presença da disfunção independe do tipo de parto (Tabela 2).

**Tabela 2:** Prevalência e comparação da disfunção sexual em mulheres no puerpério remoto em função do tipo de parto, Juazeiro do Norte, Ceará, Brasil, 2018

Tipo de parto^^*^^	Com disfunção	Sem disfunção	Valor de *p* ^^**^^
Cesariana Vaginal Total	230 (96,2%) 108 (96,4%) 338 (96,2%)	9 (3,8%) 4 (3,6%) 13 (3,8 %)	1

* Não houve dados faltantes;

** Teste do qui-quadrado.

Ao se verificar os domínios da função sexual afetados pela disfunção sexual, foram observadas correlações médias e fortes, positivas e significativas (p<0,01), evidenciando a presença de disfunção sexual em todos os domínios, com as menores pontuações nos domínios de dor e lubrificação (Tabela 3).

**Tabela 3:** Tipos de disfunção sexual identificados no puerpério remoto, Juazeiro do Norte, Ceará, Brasil, 2018

Domínios da função sexual Feminina^*^	Mediana de escore do FSFI		Valor de *p* ^**^
	Sem disfunção	Com disfunção	
Desejo Excitação Lubrificação Orgasmo Dor	3,6 3,0 2,0 2,5 1,5	1,2 1,2 1,2 1,2 1,2	<0,01 <0,01 <0,01 <0,01 <0,01

* Não houve dados faltantes;

** Teste de correlação de Pearson; FSFI - *Female Sexual Function Index.*

Outro resultado verificado foi a comparação entre satisfação sexual e disfunção sexual em puérperas a partir da utilização do teste t de Student. A média do escore da satisfação sexual das mulheres com disfunção foi estatisticamente inferior à média do escore das mulheres sem disfunção sexual (Tabela 4). Dessa forma, observou-se que a disfunção sexual diminui a satisfação sexual dessas mulheres.

**Tabela 4:** Correlação entre satisfação e disfunção sexual no puerpério remoto, Juazeiro do Norte, Ceará, Brasil, 2018

	Média da Satisfação Sexual	Valor de *p* ^^*^^
Com disfunção sexual Sem disfunção sexual	45,6 62,7	<0,01

* *Teste t de Student.*

## DISCUSSÃO

Com relação às características sociodemográficas e obstétricas da população estudada, a maioria delas correspondeu ao reportado em outras pesquisas sobre a temática^(6,24,25)^, como idade, escolaridade e paridade. Contudo, diante da complexidade e significância desse assunto, é prudente explorar outras propriedades e variáveis incluídas nesse contexto, como os aspectos subjetivos da função sexual.

Neste estudo, foi encontrada uma alta prevalência de disfunções sexuais no puerpério remoto, corroborando os resultados de uma pesquisa que utilizou o FSFI, realizada por meio de questionário online com 78 mulheres entre 45 e 180 dias de pós-parto, onde 78,2% apresentaram disfunção sexual^(11)^.

Dessa forma, ressalta-se que a função sexual feminina sofre interferência de uma complexa interação de fatores, sendo o processo de parturição um dos mais importantes, em decorrência das diversas modificações ocorridas no período gestacional, o que corrobora a alta prevalência de disfunção sexual no pós-parto^(26)^.

O tipo de parto não foi determinante para a disfunção sexual, uma vez que ela esteve presente independentemente desse fator. Esses achados coincidem com dados de uma revisão sistemática, que sustentou a irrelevância do tipo de parto sobre a função sexual feminina no pós-parto^(27)^. No entanto, embora não tenha sido verificada uma relação significativa entre as variáveis, há associações entre o parto vaginal instrumental e a lesão do esfíncter anal com um aumento nas chances de disfunção sexual no primeiro ano após o parto^(14)^.

A influência do tipo de parto sobre as alterações na função sexual também foi avaliada em outro estudo com mulheres no puerpério remoto, no qual se verificou que não houve diferenças significativas entre os grupos de mulheres submetidas ao parto vaginal quando comparadas às que realizaram cesariana^(28)^.

Os dados também indicaram em quais domínios da função sexual feminina ocorreu a disfunção, sendo que todos os domínios foram afetados. Dados semelhantes foram encontrados em um estudo com casais japoneses, no qual os escores de excitação, lubrificação, orgasmo e dor diminuíram de um a três meses após o parto^(29)^.

Os domínios de lubrificação e dor nas mulheres sem disfunção obtiveram as menores medianas no FSFI, e não houve variação nos escores medianos das mulheres com disfunção, diferentemente dos achados de uma revisão sistemática com metanálise, na qual a disfunção na fase de excitação foi mais prevalente, seguida pela fase do desejo^(30)^. Por outro lado, outra pesquisa apontou que a maior prevalência de disfunção esteve associada à presença de dispareunia^(31)^.

Os níveis de satisfação sexual das mulheres deste estudo sofreram influência significativa da disfunção sexual, impactando negativamente suas vidas no aspecto da função sexual, o que merece destaque no contexto do cuidado integral à saúde.

Os domínios da função sexual feminina são desenvolvidos de forma integrada: a excitação está ligada ao desejo, orgasmo e lubrificação, e esta afeta a dor, enquanto o orgasmo desempenha um papel vital na satisfação^(5)^. Assim, embora o envolvimento em uma atividade sexual não seja garantia de satisfação, dois domínios do funcionamento sexual, o desejo e o orgasmo, comportam-se como preditores significativos da satisfação sexual feminina e de sua parceria^(32)^.

A manutenção da função sexual e sua posterior satisfação estão entre os aspectos mais importantes da vida das mulheres, sendo relacionados à qualidade de vida^(33)^. Contudo, o aspecto da função sexual no cuidado à saúde da mulher no pós-parto é, muitas vezes, negligenciado, gerando um déficit de cuidado. Isso pode estar associado à abordagem tímida e insipiente da temática durante as consultas pré e pós-natais, seja por questões culturais, religiosas ou até mesmo por falta de conhecimento^(34,35)^.

A abordagem desse aspecto da sexualidade deve ocorrer, particularmente, na Atenção Primária à Saúde, por ser considerada a porta de entrada das redes de atenção à saúde, cuja premissa é promover ações de saúde e prevenir agravos. Assim, as ações de orientação, aconselhamento e acolhimento das demandas relacionadas às disfunções sexuais devem fazer parte do plano de cuidado dos enfermeiros, desde o pré-natal, mesmo que a mulher não manifeste diretamente a questão^(6)^.

Por fim, reforça-se que a abordagem sobre sexualidade, função, disfunção e satisfação sexual ainda é considerada um tabu, até mesmo entre profissionais de saúde, o que perpetua estereótipos culturais sobre o papel de mãe e mulher em uma sociedade estruturada sob a égide patriarcal. Por isso, é necessária a ampliação da compreensão e discussão dos múltiplos fatores envolvidos, para que se possa melhorar a atenção à saúde sexual das mulheres no puerpério.

### Limitações do estudo

As limitações deste estudo estão relacionadas à amostragem não probabilística, à ausência de análise estatística multivariada e ao fato de não ter sido considerada a visão dos profissionais de saúde das equipes da Estratégia Saúde da Família, bem como das parcerias das participantes.

### Contribuições para a área

Diante da alta prevalência de disfunção sexual no puerpério remoto encontrada neste estudo, torna-se imprescindível a atuação da equipe de saúde, especialmente do enfermeiro, na assistência ao ciclo gravídico-puerperal. A temática discutida pode trazer implicações para a prática assistencial de enfermagem, ao fornecer elementos que subsidiam a abordagem da função sexual durante a consulta de enfermagem.

Nesse contexto, o enfermeiro pode agir estruturando seus cuidados para atender às demandas identificadas, com destaque para a consulta individual, na qual são realizadas a avaliação da função sexual, a investigação dos fatores influentes sobre a função sexual, o aconselhamento sobre práticas sexuais, a conversa sobre a anatomia e fisiologia do corpo feminino e o estímulo ao autoconhecimento.

## CONCLUSÕES

Os dados obtidos indicaram uma alta prevalência de disfunção sexual no puerpério remoto. No entanto, a disfunção sexual não se correlacionou com a via de parto. A disfunção sexual esteve presente em todos os domínios da função sexual e influenciou negativamente a satisfação sexual das mulheres no puerpério remoto.

Espera-se que os dados, assim como as discussões e reflexões levantadas no estudo, subsidiem ações voltadas ao cuidado de enfermagem à saúde sexual das mulheres, considerando os aspectos da função sexual no puerpério como importantes para a qualidade de vida. Além disso, sugere-se que outras pesquisas nesse contexto sejam desenvolvidas, possibilitando conhecer o problema sob a perspectiva das parcerias sexuais e dos profissionais de saúde.
